# An Imitation-Based Treatment for Ataxic Dysarthria: A Retrospective Multiple Single-Case Study

**DOI:** 10.3390/biomedicines13071666

**Published:** 2025-07-08

**Authors:** Anna Gilioli, Sara Nordio, Zoe Ezzes, Chiara Volpato, Francesca Meneghello, Marina Zettin, Carlo Semenza, Daniela D’Imperio

**Affiliations:** 1 Neuroimaging Research Unit, Division of Neuroscience, IRCCS San Raffaele Scientific Institute, 20132 Milan, Italy; gilioli.anna@hsr.it; 2 IRCCS San Camillo Hospital, 30126 Venice, Italy; 3 Memory and Aging Center, Department of Neurology, University of California, San Francisco, CA 94158, USA; 4 Department of Psychology, University of Torino, 10124 Turin, Italy; 5 Centro Puzzle, 10137 Turin, Italy; 6 Padova Neuroscience Center, University of Padova, 35131 Padova, Italy

**Keywords:** ataxic dysarthria, cerebellar ataxia, action observation therapy, imitation, speech and language therapy

## Abstract

**Background/Objectives:** Ataxic dysarthria is a speech disorder characterized by the impaired coordination of movement due to cerebellar dysfunction. Despite its clinical relevance, few studies have explored its rehabilitation. This study aimed to evaluate the applicability of IMITAF, an adaptive computer-based clinical treatment protocol originally developed to target aphasia with a novel population comprising individuals with ataxic dysarthria. The approach leverages principles of procedural motor learning. **Methods:** Ten patients with ataxic dysarthria due to neurodegenerative disease were retrospectively studied. All patients received approximately one month of speech–language (SL) treatment. Among them, (1) three patients (LL, MD, and BoA) adjunctively received the IMITAF treatment, forming the experimental group, and (2) the remaining seven patients did not receive IMITAF, serving as the control group. Dysarthria was assessed using the “Protocollo di Valutazione Disartria e Disfonia” (PVDD). The applicability of IMITAF was assessed through within-session performance and by direct single-case comparisons of total PVDD scores pre- and post-treatment. Additionally, multiple single-case Crawford analyses were conducted using PVDD scores and subscores to compare trained (i.e., directly targeted) and untrained abilities between the experimental and control groups **Results:** Patients in the IMITAF group showed improvements during exercises, with further increases in total PVDD scores post-treatment. Two patients (LL and BoA) showed significant gains, while MD’s scores remained stable. Compared to the control group, all three experimental patients demonstrated measurable improvements in trained core deficits associated with dysarthria, including phonation, articulation, intelligibility, and prosody (as assessed by PVDD). **Conclusions:** These findings suggest that IMITAF may offer therapeutic benefits for patients with ataxic dysarthria. By engaging a cortico-subcortical network involved in procedural motor learning, IMITAF may help mitigate speech deficits resulting from cerebellar dysfunction. This preliminary evidence supports the potential of IMITAF as a promising adjunctive tool in the rehabilitation of ataxic dysarthria.

## 1. Introduction

Ataxic dysarthria is a hallmark symptom of cerebellar dysfunction and is characterized by the impaired coordination of speech movement sequences. Although it has a relatively rare incidence, it arises from diverse etiologies, including neurodegenerative disease (e.g., hereditary, as in Friedreich’s ataxia and spinocerebellar ataxia [1]) and acquired conditions (e.g., stroke or encephalic infections [2,3]). Notably, early-onset cerebellar degeneration [4] and the associated ataxic dysarthria occur in young adults, highlighting the disorder’s clinical relevance within chronic, progressive neurological conditions [2]. The core speech deficits in ataxic dysarthria are due to coordination and timing impairments in speech production [5,6,7], specifically manifesting as the following [8]: (i) articulatory difficulties (e.g., inaccuracy, phonetic distortions, irregular breakdowns); (ii) prosodic impairment (e.g., excess and equal stress, phoneme prolongations, slow rate of speech); and (iii) phonatory difficulties (e.g., disrupted speech–breath coordination, excessive loudness variation). The functional consequence of these impairments is a reduction in speech intelligibility [7], which negatively affects communication efficacy and thus the quality of life [9,10].

Given the considerable impact of progressive dysarthria, even in young adults, targeted rehabilitation is critically important [10,11]. Both pharmacological and behavioral interventions have been proposed for managing progressive dysarthria in neurodegenerative diseases [12,13]. Speech–language therapies typically aim to enhance intelligibility and communication effectiveness by targeting the respiratory, phonatory, articulatory, and prosodic domains [14,15]. Within these domains, articulatory inaccuracy is a primary perceptual marker and, thus, often serves as the main therapeutic target [8]. Prosody is another key target, with several treatment protocols emphasizing the modulation of pitch, intensity, and duration in order to improve the accuracy of stressed syllables [16]. Improvements in prosodic control have been shown to enhance both the naturalness and intelligibility of speech in individuals with ataxic dysarthria [17,18]. Additionally, orofacial motor abilities, such as lip protrusion, tongue mobility, and jaw control, are sometimes addressed in treatment [19], although the efficacy of directly targeting the speech musculature remains debated [20].

The Lee Silverman Voice Treatment (LSVT), originally developed for hypokinetic dysarthria in Parkinson’s disease [21], has shown promise in treating ataxic dysarthria [22]. LSVT focuses on increasing vocal loudness and improving vocal quality. Its application in ataxic dysarthria has been associated with gains in articulatory accuracy, phonation, speech intelligibility, and overall communicative abilities [23]. Further, LSVT has demonstrated positive effects on patients’ quality of life [10], suggesting that objective measures of improvement are actually functionally meaningful.

Other behavioral strategies support speech rehabilitation enhancing sensorimotor function. This includes posture optimization, motor strengthening, and motor control [15]. For instance, techniques such as phonetic placement cues, hyperarticulation, and rate modification have also been employed to improve intelligibility. For example, rhythmic cueing during reading tasks has helped with regulating speech patterns in those with mixed spastic–ataxic dysarthria [18,24]. Additionally, biofeedback tools providing real-time auditory and visual feedback on loudness and pitch have similarly been effective in improving both the intelligibility and naturalness of speech, facilitated by enhanced awareness and self-monitoring [1,25].

Additional interventions have focused on optimizing communicative effectiveness by manipulating environment variables (e.g., minimizing background noise, ensuring face-to-face interaction) or by incorporating nonverbal modalities such as facial expressions, gestures, or augmentative communication devices [15]. Moreover, group therapy could also be beneficial, leveraging socially meaningful contexts to improve communication skills [26]. Preliminary evidence also supports the use of noninvasive brain stimulation techniques, though the current literature is limited [27]. Despite these promising interventions, the rarity and heterogeneity of ataxic dysarthria present challenges to conducting large-scale homogeneous studies. Consequently, the generalizability of the existing findings remains limited [10,28,29].

Current evidence of the benefits of rehabilitation in neurodegenerative ataxic dysarthria primarily derives from small cohorts and case studies, with generally modest improvements in intelligibility and speech naturalness [1,23].

Furthermore, such improvements often rely heavily on continuous external feedback to be generalizable in terms of daily communication [18,24] and are influenced by heterogeneity in individual patient characteristics [1,25]. Given these constraints, a more individualized research design may be warranted. A multiple case study design, as opposed to large-group trials, is better suited to highlighting individual variations within this heterogeneous population rather than focusing on average effects. Additionally, current research does not adequately address the treatment of neurodegenerative ataxic dysarthria from a motor learning perspective. The utility of imitation for the rehabilitation of gross motor functions has been well documented, particularly in interventions such as Action Observation Therapy (AOT) [30]. In speech rehabilitation, imitation has also proven beneficial, for instance, in speech entrainment and script training for those with nonfluent aphasia or apraxia of speech and in the IMITATE program, which has shown success across a spectrum of aphasia types, severity, and degree of motor speech involvement [31,32].

The simultaneous observation and imitation of a speaker’s mouth during oral communication engages a frontoparietal neural network [33], and several studies have implicated the role of the cerebellum and subcortical structures in imitative actions, particularly for vocal learning [3,34].

Moreover, the neural circuits engaged in the imitation of oral motor functions partially overlap with language-related areas when linguistic content is involved, supporting naming and repetition abilities [35].

Building on these principles, the researchers developed IMITAF, an innovative, adaptive, computerized program centered on the observation and imitation of everyday speech actions [32]. Grounded in motor learning theory, IMITAF facilitates speech recovery by reinforcing procedural motor patterns through structured, repetitive practice [31]. Its use has shown positive effects in post-stroke patients with varying degrees of aphasia, yielding improvement in word retrieval, naming, and speech repetition [32,36]. These findings suggest that IMITAF may be a valuable tool for training procedural speech motor skills.

To date, IMITAF has not been systematically tested in individuals with dysarthria, but its underlying principles are well-aligned with established articulation training approaches used in other neurodegenerative conditions [22,37]. IMITAF targets articulation, prosody, phonation, and speech intelligibility through structured repetitive and adaptive exercises, which are critical for effective motor learning. Repetitive training reinforces motor memory and promotes the consolidation of the procedural motor patterns necessary for speech.

Given the shared cortico-subcortical networks, particularly involving the cerebellum, between motor and language functions [29], IMITAF exercises using meaningful sentence-level stimuli may help enhance motor programming for speech. Within this framework, by using an adaptive task tailored to the patients’ abilities, IMITAF may serve as an adjunctive tool to support oral motor actions and improve speech proficiency in patients with cerebellar diseases resulting in ataxic dysarthria.

This preliminary study aimed to explore the effectiveness of IMITAF in treating ataxic dysarthria, using a multiple single-case analysis. Specifically, three patients with neurodegenerative ataxic dysarthria received IMITAF in addition to standard therapy and were compared to seven control patients who underwent conventional speech–language (SL) therapy alone. The study objectives were twofold:

(i) To evaluate the applicability of the imitation-based IMITAF treatment for individuals with ataxic dysarthria in clinical settings.

(ii) To assess the therapeutic benefits of IMITAF as an adjunctive rehabilitation protocol to traditional SL therapy. Given the progressive nature of the underlying neurodegenerative conditions, the primary therapeutic aim was to slow the progression of speech decline and maintain communicative function over time.

## 2. Materials and Methods

### 2.1. Participants

This study retrospectively enrolled ten patients with neurodegenerative cerebellar ataxia who were hospitalized at IRCCS San Camillo Hospital in Venice (Italy) between January 2015 and December 2022. The following inclusion/exclusion criteria were applied to ensure a consistent sample: (1) a cerebellar etiology of deficits, with no reported influence from other neurological or psychiatric disorders or new clinical symptoms; (2) a confirmed diagnosis of ataxia with dysarthria, based on independent evaluations conducted by both an expert neurologist and a speech–language pathologist trained in the “Protocollo di Valutazione Disartria e Disfonia” (PVDD) [38]; and (3) participation in a structured SL treatment program with both pre- and post-treatment dysarthria evaluations. Eligible patients were contacted and asked to provide informed consent. This study was conducted in accordance with the Declaration of Helsinki and was approved by the local ethics committee for clinical research.

In this retrospective cohort of ten patients, all participants underwent standard SL treatment for approximately one month, consisting of daily one-hour sessions, four days per week. Among these, three patients identified as LL, MD, and BoA (forming the experimental group) adjunctively received the computerized IMITAF treatment, consisting of 20 daily one-hour sessions. At the time of IMITAF treatment initiation, BoA was not receiving any pharmacological treatment, and, as with the other enrolled patients, the maintenance of performance was desirable.

We therefore retrospectively selected two groups of patients: seven patients receiving only routine SL rehabilitation (i.e., the control group) and three patients receiving routine SL supplemented with IMITAF treatment (i.e., the experimental group). Available clinical data were collected to describe the patients and monitor changes in dysarthria severity.

### 2.2. Rehabilitation Protocols

#### 2.2.1. Routine SL Treatment

Routine rehabilitation was administered to all ten patients by a speech–language pathologist, either individually and/or in groups (with 1 h sessions four or five times per week), tailored to patients’ clinical needs. Treatment lasted approximately one month (range: 18–34 days; mean = 23.22 ± 5.86). Exercises were tailored according to patients’ impairments by adjusting task difficulty (e.g., number of words, syntactic complexity, and diversity of phonotactic structures). These exercises were then practiced in conversational settings to promote generalization and simulate an ecologically valid setting. The training involved combined respiratory and phonatory exercises, orofacial muscle exercises, articulation exercises, prosody exercises, and the development of self-monitoring skills to enhance independent strategy use ([37], see Table S1 in Supplementary Materials for an overview of the exercises). Throughout each treatment session, the speech–language pathologist provided feedback on strategy use, employing well-established communication techniques such as fading, shaping, modeling, prompting, reinforcement, and error Correction [39,40].

#### 2.2.2. IMITAF Treatment

Patients in the experimental group completed the above-mentioned routine SL training with the addition of the IMITAF treatment for 20 one-hour daily sessions, with 4 to 5 sessions per week, following the original IMITAF protocol [32].

IMITAF sessions were performed on a computer under the guidance and supervision of an SL therapist. It consists of a video-based intervention delivered through a series of clips that open in VLC Media Player. During each session, patients watched and listened to videos of six different actors, each with different Italian accents (three males and three females of various ages), speaking aloud each stimulus (i.e., words or sentences). Actors were shown from the waist up to focus patients’ attention on facial movements and to prepare for the future training of turn-taking strategies. Each stimulus was repeated six times, followed by a plain, black screen displaying text asking the patients to repeat what they had just heard. To minimize learning effects from repeating the same stimuli, the stimuli were presented in random order within each difficulty level.

The IMITAF protocol includes 16 levels progressing in difficulty adapted to varying severities of speech impairment [32]. Levels range from disyllabic words to complex sentences, beginning with polysyllabic words and simple sentences, increasing incrementally in complexity. The final level combines elements of previous levels (see [32] for further details). All three participants started IMITAF treatment at level 9 (i.e., simple sentences) as their initial PVDD speech evaluations classified their dysarthria severity as mild and they demonstrated the ability to accurately repeat disyllabic and polysyllabic words during IMITAF treatment.

Importantly, the stimuli are ecologically valid, derived from connected speech with normal prosody, and spoken by multiple distinct voices [32]. The IMITAF protocol is individualized by the speech–language therapist, who adjusts structured exercises to optimize treatment outcomes. If a patient does not achieve a level of accuracy—demonstrated by intelligibility without repetition or effort for most sentences—tasks are modified accordingly. For example, changing a sentence’s communicative goal (e.g., converting a statement to question) aids prosodic control, while adjusting breath points supports respiratory and phonatory function. Stimulus volume helps regulate loudness dynamics, and emphasizing key words enhances speech naturalness. Targeted cues, such as exaggerated articulatory movements or slowed speech, improve clarity. Additionally, the SL therapist provides verbal, visual, or tactile cues as feedback to support correct sentence productions and facilitate self-monitoring, a consistent approach used in routine SL treatment. At the end of each session, the clinician initiates a brief conversation to encourage the generalization of strategies to natural communication settings.

During the sessions, patients viewed the videos and received feedback from the therapist as needed. Once the mastery of the current difficulty level was achieved, indicated by the patient’s ability to produce sentences clearly and intelligibly without repetition, and the patient could generalize their skills to broader contexts (as described in [32]), the SL therapist progressed the patient to the next level in the hierarchy.

### 2.3. Available Functional and Cognitive Assessments

We collected data on the examination of functional abilities using the Functional Independence Measure (FIM) [41] and the Barthel Index [42], which assess both motor and cognitive functioning. We collected data from neuropsychological tests for verbal fluency ability (i.e., phonemic and semantic fluency) [43,44] and verbal short-term and working memory (i.e., digit span forward and digit span backward, respectively [45]). In the experimental group, a neuropsychological battery of general cognitive functioning (Addenbrooke’s Cognitive Examination-Revised, ACE-R; [46]) was completed but was missing in the control group. Since this is a retrospective study, we reported on only the available, previously completed, and administered cognitive assessments.

#### Measuring Ataxic Dysarthria

In all patients, dysarthria severity was evaluated before and after treatment using the PVDD, which assesses various speech parameters including intelligibility (i.e., level of understanding of speech), respiration (i.e., duration of the /s/ sound), phonation (i.e., duration of the /a/ sound and perceived vocal fatigue), diadochokinesis (i.e., accuracy in repeating single and multisyllabic sounds ), orofacial muscle function (i.e., evaluation of each muscle’s strength, range of motion, symmetry, etc.), prosody (i.e., rhythm, rate, intonation, and stress of specific words and phrases), and articulation (i.e., word repetition and reading of a text). These parameters were evaluated through systematic tasks and exercises to ensure the consistent quantification of deficits (for all details see [38]). Moreover, the test includes a section that evaluates general speech intelligibility through spontaneous conversation. The PVDD was available both pre- and post-rehabilitation for all patients, and it was used as the primary measure of dysarthria severity in the statistical analysis. Given that the interventions were conducted in a clinical setting, the PVDD was typically administered by a different SL therapist than the one delivering the IMITAF treatment. However, in the case of patients receiving only routine SL therapy, this separation of roles occurred less frequently.

The total PVDD score comprises 35 items (each with scores from 1 = severely impaired to 4 = spared ability, yielding a total score range of 35–140) [38]. In addition to the total score, we developed an ad hoc categorization of three PVDD subparts to distinguish their abilities based on their relevance to the treatment targets, as implemented in previous rehabilitation studies [47]:

(I) “Trained”, comprising 10 items from the subscales of intelligibility, prosody, articulation, and phonation (score range: 10–40), representing primary outcomes directly targeted by the treatment.

(II) “Near Untrained”, comprising 9 items from the respiration and diadochokinesis subscales (score range: 9–36), representing secondary abilities potentially influenced by the treatment.

(III) “Far Untrained”, comprising 16 items from the orofacial muscle function subscale (score range: 16–64), with minimal expected treatment-related effects.

This categorization allowed for a more nuanced examination of rehabilitation outcomes, enabling us to explore the effects of IMITAF based on whether the targeted abilities were directly trained, proximally related, or unrelated to the intervention.

### 2.4. Behavioral Analysis

In order to check differences between groups, the following was conducted:

- The two groups were compared in terms of available demographic, clinical, and cognitive data as well as baseline total PVDD scores. Mann–Whitney tests were performed for nonparametric variables; a chi-square test was used to assess the group differences in proportions (e.g., gender).

Furthermore, to investigate the use of IMITAF, the analyses consisted of the following steps:

- The applicability of IMITAF was evaluated for each participant’s progress on the difficulty levels achieved during IMITAF sessions by means of C-tests [48] to analyze trends in changes in performance during IMITAF treatment, examining within-participant progress throughout the treatment.
- The possible benefits of IMITAF were investigated first through general improvements, by comparing each patient’s pre- and post-treatment total PVDD scores by means of Sign Tests for repeated nonparametric data [49]. Secondly, to understand the possible effects of IMITAF as an adjunctive training regimen to routine SL training, each experimental participant was compared to the control group by means of adapted t-tests for single-case Crawford analysis (i.e., DIFFLIMS.EXE [50]). The effects of rehabilitation were evaluated as the difference between the pre- and post-evaluation measures of dysarthria. DIFFLIMS.EXE enables a comparison of the effects observed in each experimental patient to those in a control sample. This test was repeated on the total PVDD score as well as the adapted scores of PVDD subparts.

All analyses were conducted using R through JAMOVI (version 2.3) [51] and R Studio (version 2023) [52]. Statistical significance was set at a *p*-value < 0.05.

## 3. Results

### 3.1. Comparability Between Groups

Demographic, clinical, and cognitive variables were compared between the experimental and control groups. The two groups were also compared in terms of baseline speech abilities as measured by the total PVDD score. Furthermore, given the retrospective framework, the different treatment durations were compared to verify consistency across groups.

The control group did not statistically significantly differ from the experimental group in terms of demographic factors such as age (t = −0.640, *p* = 0.540; experimental group mean = 48.33 ± 4.73, control group mean = 54.57 ± 16.09) and education (t = 0.404, *p* = 0.697; experimental group mean = 10.43 ± 3.36, control group mean = 10.43 ± 3.36), but they did differ in terms of gender (chi-squared= 4.29, *p* = 0.038; experimental group male = 100%, control group 28.57% male). Demographic data and statistics for the entire sample are reported in Table 1.

When assessed for their functional and cognitive abilities (Table 2), no differences were observed for dysarthria in the total PVDD score (t = 1.084, *p* = 0.310; experimental group mean = 122 ± 13, control group mean = 113.29 ± 11.16) nor for functional evaluation in the FIM (t = 0.174, *p* = 0.866; experimental group mean = 80.67 ± 5.69, control group mean = 79.14 ± 14.29) and the Barthel Index (t = 0.917, *p* = 0.386; experimental group mean = 65 ± 5, control group mean = 55 ± 18.03). No significant between-group differences were noted for other cognitive measures (i.e., verbal fluency tests, digit tests). In addition, we report that the three patients within the experimental groups had an ACE-R score within normal limits (i.e., equivalent scores—ES ≥ 1), highlighting preserved cognitive functioning: LL total score = 84.89, ES = 3; MD total score = 90.29, ES = 4; and BoA total score = 92.89, ES = 4.

Finally, the different treatment durations did not show any significant differences (t = −6, *p* = 0.48; experimental group mean = 20 ± 0, control group mean = 24.8 ± 6.77).

### 3.2. Applicability of IMITAF

**Figure 1:**
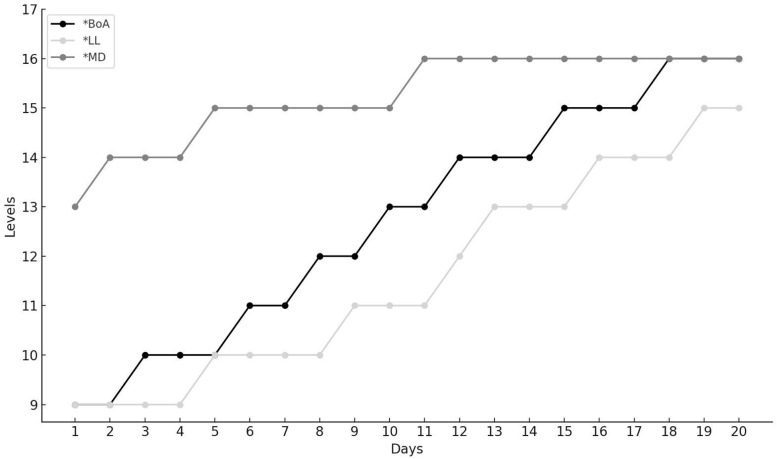
IMITAF training with personalized session level with respect to speech pattern of participant (* *p* < 0.0001).

All three participants performed at higher difficulty levels in the IMITAF exercises by the end of treatment relative to their starting levels, indicating task-specific improvements. These gains are demonstrated by means of C-tests for LL (C = 0.964, z = 4.541, *p* < 0.0001), MD (C = 0.905, z = 4.260, *p* < 0.0001), and BoA (C = 0.968, z = 4.558, *p* < 0.0001) (Figure 1).

### 3.3. Benefits of IMITAF

The direct single-case comparisons between pre- and post-IMITAF on total PVDD scores demonstrated improved post-treatment outcomes for LL (*p* = 0.0002; pre-total = 107, post-total = 120) and BoA (*p* = 0.016; pre-total = 130, post-total = 137), while scores remained stable for MD (*p* > 0.05; pre-total = 129, post-total = 130).

Using single-case Crawford comparisons against the control group, statistically significant differences are reported in the total PVDD score (control group pre-sum mean = 113.29 ± 11.16; post-sum mean = 115.43 ± 11.66) for LL (t = −2.697 *p* = 0.036; pre-sum = 107, post-sum = 120). Statistically significant differences are also reported for trained abilities (control group pre-sum mean = 30.86 ± 4.95; post-sum mean = 31.43 ± 4.50) for LL (t = −5.227 *p* = 0.040; pre-sum = 33, post-sum = 39), MD (t = −3352, *p* = 0.014; pre-sum = 34, post-sum = 38), and BoA (t = −2.606, *p* = 0.040; pre-sum= 35, post-sum = 38) (Table 3). No other differences were observed.

## 4. Discussion

This pilot study is the first to apply the computerized IMITAF treatment to patients with ataxic dysarthria resulting from cerebellar involvement. IMITAF has previously been used to improve word-finding difficulties in those with aphasia [31,32] as a systematized, intensive, and personalized program focused on speech action imitation, grounded in motor learning principles, and underscoring the crucial role of the cerebellum [3].

We hypothesized that IMITAF could be particularly suitable for addressing ataxic dysarthria as this condition primarily involves peripheral speech deficits related to motor control and coordination. The use of standardized materials with progressively increasing difficulty enables tailored interventions, adapting them to each patient’s specific abilities and promoting gradual improvements in articulatory accuracy and prosody.

The primary aim of the present study was to explore the applicability of IMITAF for ataxic dysarthria. The analysis of each participant’s performance during IMITAF sessions revealed that all participants showed improvement, as indicated by their ability to achieve higher difficulty levels compared to their initial starting point. In fact, the inherent learning principle of this protocol is based on the possibility of gradually increasing the level of difficulty, enabling patients to improve incrementally as they master each level [32]. Beyond just initial dysarthria severity, the treatment’s starting points are also personalized to address individual speech difficulties, with various adaptations available to address the heterogeneity in speech features observed among patients with ataxic dysarthria.

To further ensure some potential benefits of the IMITAF protocol in patients with ataxic dysarthria, PVDD scores were compared before and after training using a multiple single-case analysis. Both BoA and LL showed improvements in their total PVDD scores, while MD’s performance remained consistent throughout the treatment duration, despite initial signs of improvement. However, given that the etiology of MD’s motor speech impairment is degenerative, generally consistent performance can still be indicative of the prevention of further decline. Furthermore, comparison analyses with the control group show specific improvements. We may suppose that some effects of IMITAF in the training of defects are due to ataxic dysarthria.

In addition to exploring the role of IMITAF as an adjunctive therapy to conventional SL training, we also compared the three experimental patients (who received combined IMITAF and SL treatment) to a control group that received only conventional SL treatment. The groups were comparable in terms of age, education, and disease duration (Table 1); functional and neuropsychological performance (Table 2); and baseline dysarthria severity, with only minimal differences in sex distribution. Subsequently, the multiple single-case Crawford comparison showed that in comparison to the control group, LL performed better on the total PVDD score, and all experimental patients demonstrated greater improvement in “Trained” scores (from PVDD) than the control patients. These findings suggest that pre- to post-training changes were more significant when SL was combined with IMITAF than with SL alone, particularly in the directly targeted trained abilities. The combination of SL and IMITAF resulted in more pronounced improvements in the core deficits of ataxic dysarthria, which represent key targets of rehabilitation. For all three experimental patients, IMITAF appeared to specifically provide better treatment outcomes for intelligibility, phonation, prosody, and articulation than the SL protocol alone, suggesting that IMITAF may represent a viable, tailored rehabilitation strategy for ataxic dysarthria.

Ataxic dysarthria is a heterogeneous disorder with variations in clinical presentation, allowing for the identification of different subgroups based on the severity of motor involvement and speech impairments [53]. Studies have suggested that patients may respond differently to rehabilitation interventions, highlighting the need for personalized treatment approaches [54]. Existing therapy programs in ataxic dysarthria focus on articulatory inaccuracy [23] and prosody [16,17], both of which are important for speech intelligibility [18]. Notably, in our results, the patients who received IMITAF significantly improved in these domains, classified as “Trained”, showing that the treatment may specifically support these abilities (see Table 3 for data). Moreover, our patients improved in phonation (part of “Trained” abilities). Therefore, participating in an intensive 4-week speech therapy program may help participants build endurance for regular, demanding practice, potentially reducing speaking effort and, consequently, the degree of speech-related fatigue [55], as assessed in items related to phonation in PVDD.

The cerebellum plays a crucial role in articulatory coordination and timing patterns; thus, its damage can result in speech timing deficits and irregular durational parameters [56]. These impairments may be key targets for the rehabilitation of ataxic dysarthria. We hypothesize that the repeated imitation of sentence-level stimuli with appropriate intonation during IMITAF training could engage motor learning processes by engaging the broad cortico-subcortical neural network, strengthening articulation, prosody, and phonation [57]. Moreover, the preserved cognitive functions observed in our patients, particularly verbal short-term memory and fluency, may support the consolidation of learned skills over time, essential for successful engagement in therapy. These results suggest that this training may help maintain abilities and manage dysarthria, potentially delaying the loss of intelligible speech and improving patients’ quality of life during their disease course. To understand the potential benefits of IMITAF, it is important to highlight the clinical contribution of therapist feedback on utterance production during the IMITAF imitation sessions. The crucial role of feedback in speech production has already been emphasized in the literature on ataxic dysarthria, where difficulties in predicting the execution of motor actions are closely linked to the prosodic deficits exhibited by these patients [6]. Indeed, the therapist’s feedback throughout the IMITAF sessions can support this function, in addition to the videos themselves, by helping patients plan and execute their actions, supporting self-monitoring.

We found that IMITAF focuses on controlling stress patterns by training intonation flexibility through personalized exercises. Notably, articulation and prosody are two of the most affected parameters in ataxic dysarthria, so IMITAF may help support the residual function of speech motor movements and intonation. Furthermore, this treatment may encourage patients to monitor and improve their own articulatory accuracy: the clinician provides multiple opportunities for the patient to correctly repeat the sentences and offers corrective feedback for each production. Similarly, SL treatment also contributes to improving the patients’ ability to self-monitor their speech abilities, supporting their overall rehabilitation.

We may take into consideration that IMITAF represents a new approach to treating ataxic dysarthria by targeting imitation circuits to support existing and re-learned impaired oral motor actions. The cerebellum is involved in the imitation circuit [34], as it seems to be affected in our group (Table 1). However, in ataxic dysarthria, oral motor learning can remain intact depending on the nature of the task, with the procedural learning system seemingly relatively preserved [23,58]. This suggests that procedural motor memory could provide a viable route for delivering effective speech therapy to these patients, engaging the preserved procedural system through therapeutic tasks such as repeated sentence imitation in IMITAF. In fact, the gradual presentation of increasingly difficult stimuli and responses encourages the procedural memory system to learn motor sequences [57,58].

These results are further supported by the involvement of the cerebellum in the control of both motor and nonmotor aspects. The cerebellum is integrated into a broad cortico-subcortical neural network that connects to speech and language areas [57]. Through this network, the cerebellum supports two main functions, motor control and coordination, particularly in relation to oral motor actions, frontal functions, and general cognition [57]. Our three experimental patients exhibited some cerebellar damage, but they did not show deficits in verbal fluency, which may instead involve a broader neuronal network beyond the cerebellum, potentially supporting rehabilitation for patients with ataxic dysarthria. IMITAF’s approach is based on the assumption of activating a broad neuronal circuit for speech through the observation and imitation of “acts of speaking,” similar to previous studies where aphasic patients improved compared to a control group using static images [31]. In this context, IMITAF may be particularly suitable for ataxic dysarthria patients in a training program focused on the peripheral aspects of speech, such as phonation, articulation, and prosody, with potential support for speech abilities. The findings of this pilot retrospective study may provide a starting point for understanding ataxic dysarthria and its rehabilitation.

Despite the promising results, some limitations must be acknowledged. First, this study was conducted within a single clinical institution, which may limit the generalizability of the findings. Given the heterogeneous and rare nature of cerebellar ataxia, a multicenter design would have been particularly beneficial to enhance the diversity and representativeness of the patient sample. However, the present study was conceived as a retrospective, exploratory investigation primarily aimed at assessing the feasibility and potential of the IMITAF protocol for this specific population. At the time of data collection, the IMITAF intervention required specific clinician training and materials. These methodological and practical considerations, along with the need to first evaluate the protocol’s clinical applicability, explain the single-center approach. The retrospective nature of our study did not allow for a fully controlled design (i.e., blinded assessment, clinical treatment feedback, and length). The challenges inherent in designing and conducting robust, large-scale trials underscore the need for further research to develop standardized, evidence-based protocols tailored to the unique characteristics of ataxic dysarthria. Nevertheless, the positive preliminary findings support the rationale for future prospective, multicenter studies designed to expand on these results and evaluate IMITAF’s effectiveness in broader and more diverse clinical settings. A multicenter design would allow for greater clinical and demographic variability, facilitate subgroup analyses, and improve the external validity of the findings.

Second, the clinical heterogeneity of the included patients, particularly in terms of lesion site and diagnosis, could have influenced treatment outcomes. Although all participants were diagnosed with ataxic dysarthria and presented with cerebellar involvement, the underlying etiologies differed (e.g., degenerative versus vascular), and so did the neuroanatomical profiles. These differences may affect both baseline severity and responsiveness to treatment. For example, one of the experimental patients (MD), who had a degenerative etiology, showed stabilization rather than improvement—potentially reflecting a different pathophysiological trajectory. While our findings suggest that IMITAF can benefit a range of ataxic profiles, future studies should examine whether specific subtypes respond more favorably to the intervention. Stratifying patients by lesion characteristics and etiology may provide valuable insights into treatment mechanisms and inform tailored rehabilitation approaches.

Third, the composition of the experimental and control groups differed in certain respects, particularly with regard to gender distribution and cognitive profile. The experimental group consisted of three male patients with normal-range ACE-R scores, whereas the control group included both male and female patients, and ACE-R data were unavailable. This discrepancy reflects the retrospective nature of this study and the limited availability of cognitive assessments for all participants. While we attempted to match the groups in terms of demographic variables (age, education, disease duration) and functional measures (FIM and Barthel Index), the absence of standardized neuropsychological data for the control group limits our ability to fully account for cognitive influences on therapy outcomes. We suppose that cognitive functions—especially working memory, attention, and executive abilities—might play a critical role in the acquisition and retention of speech motor skills, and differences in these domains could have contributed to outcome variability. We therefore suggest the use of the IMITAF protocol with relatively preserved cognitive functions, similar to our three experimental cases, and when the target of rehabilitation is ataxic dysarthria resulting from cerebellar involvement.

However, this study demonstrated the effectiveness of applying IMITAF in a naturalistic clinical setting. Moreover, the results highlighted the promising benefits of IMITAF as an easily applicable clinical intervention, providing preliminary data to support future investigations. In fact, a key component of IMITAF is its focus on individualizing and adapting the treatment to each patient’s needs, and demonstrating its application through single-case analyses is invaluable for understanding how different patients may respond to this treatment, identifying its potential effects on dysarthria deficits in improving and maintaining abilities in patients with neurodegenerative etiologies.

In conclusion, the IMITAF protocol, with its adaptive approach tailored to individual patients, appears to be effective in a novel population of patients with ataxic cerebellar dysarthria. Based on its previous application to patients with aphasia in naming tasks [32], we support the idea that IMITAF can be tailored for individuals with diverse profiles [32]. It will be important for clinicians to consider the specific characteristics of each patient’s dysarthria and disease profile when developing an effective, personalized rehabilitation program.

## 5. Conclusions

The present pilot study serves as a starting point for understanding the clinical management of ataxic dysarthria in individuals with degenerative cerebellar etiologies. Given the nature of degenerative diseases, clinical management should include efforts to improve present functioning, maintain current abilities, counteract the progression of deficits, train self-monitoring skills, and teach compensatory strategies. Our protocol provides preliminary support for IMITAF, an adaptive, individualized, computer-based therapy tool, in treating patients with neurodegenerative ataxic dysarthria. In comparison to traditional SL intervention alone, IMITAF may provide additional therapeutic benefits in the speech domains impacted by ataxic dysarthria. Our study suggests that using an approach such as IMITAF may help to preserve and improve speech abilities by recruiting preserved procedural memory, making it an effective tool for those with ataxic dysarthria from cerebellar involvement who may have relatively preserved cognitive functioning.

## Figures and Tables

**Table 1:** Patients’ demographics and clinical features related to ataxic dysarthria. BoA was unexposed to pharmacological treatment for inflammation at the time of IMITAF treatment. Statistical comparisons between the experimental and control groups are shown by *p*-values (* indicates *p*-value < 0.05). Abbreviations: GHS = Gordon Holmes Syndrome; SCA = spinocerebellar ataxia (type of SCA is indicated by a number when available); CS = Cerebellar Syndrome; FRDA = Friedreich Ataxia; RH = right; LH = left; st-Vs = supratentorial Ventricles; Fg = Focal gliosis; Mb = Midbrain; P = Pons; Mo = Medulla oblongata; bl-FP = bilateral frontoparietal; VH = Vermal Hypothropy; SS= Supra-Sylvian; FWM = Frontal White Matter.

Patient	Sex	Age	Education	Years of Disease	Diagnosis	Lesion
Experimental group						
LL	M	52	13	21	GHS	Cerebellar hypoplasia with inferior vermis involvement.
MD	M	43	8	21	SCA	Cerebellar atrophy.
BoA	M	50	13	0	CS—inflammatory origin	Altered signals in Mb tegmentum and RH cerebellar hemisphere.
Control group						
LP	M	54	8	5	Ataxia unspecified	Enlargement of st-Vs, IV ventricle, and sulci. Altered signals in Mb-P-Mo, IV ventricle, and LH cerebellum. Focal gliosis in bl-FP WH.
CM	F	29	13	4	SCA2	Cerebellar atrophy.
MG	F	51	8	8	SCA2	Cerebellar atrophy.
SA	M	68	5	19	SCA	Cerebellar atrophy.
BA	F	67	13	32	FRDA	Enlargement of cerebellar sulci. VH.
MS	F	73	13	3	SCA2	Very small post-ischemic lesions in RH SS and LH FWM.
MA	F	40	13	24	SCA	Cerebellar atrophy.
*p*-value	0.038 *	0.517	0.794	1.00		

**Table 2:** Functional abilities assessed by FIM and Barthel Index and available neuropsychological data at baseline (scores corrected for age and educational level). FIM score range is 18–126; Barthel Index score range is 0–100; cut-off scores for pathological performance are as follows: Phonemic fluency < 17; Semantic fluency < 25; Digit span forward < 4.26; Digit span backward < 2.65. Na = Not available. Pathologic score is indicated in bold.

Patient	FIM	Barthel Index	Phonemic Fluency	Semantic Fluency	Verbal Short-Term Memory (Digit Span Forward)	Verbal Working Memory (Digit Span Backward)
Experimental group						
LL	79	60	28.7	30.7	5.83	3.79
MD	76	70	37.8	37.6	4.89	3.96
BoA	87	65	20.5	40.5	4.75	3.71
Control group						
LP	82	60	33.9	46.9	4.96	4.02
CM	86	60	24.4	35.4	4.44	3.42
MG	86	60	Na	Na	Na	Na
SA	82	50	21.3	27.6	4.39	3.53
BA	47	20	Na	35.4	6.02	**1.97**
MS	86	55	Na	Na	Na	Na
MA	85	80	Na	Na	Na	Na
*p*-value	0.817	0.198	1.00	0.858	0.857	0.400

**Table 3:** Data for the control (mean ± standard deviation) and experimental (raw data) groups are reported at pre- and post-treatment and in delta (**Δ**) scores (post- minus pre-treatment data). * = significant comparisons from *t*-tests for single-case Crawford analysis.

Treatments			
	Pre	Post	Δ Post–Pre
Control group			
PVD total (max = 140)	112.5 ± 11.11	114.67 ± 12.58	+2.17
Trained (max = 40)	7.88 ± 3.69	7.92 ± 3.84	+0.04
Near Untrained (max = 36)	10.58 ± 4.62	11.67 ± 5.03	+1.09
Far Untrained (max = 64)	59.83 ± 4.36	59.67 ± 4.84	−0.16
Experimental group			
LL			
PVD total (max = 140)	107	120	+13 *
Trained (max = 40)	33	39	+6 *
Near Untrained (max = 36)	22	29	+7
Far Untrained (max = 64)	52	52	0
MD			
PVD total (max = 140)	129	130	+1
Trained (max = 40)	34	38	+4 *
Near Untrained (max = 36)	31	28	−3
Far Untrained (max = 64)	64	64	0
BoA			
PVD total (max = 140)	130	137	+7
Trained (max = 40)	35	38	+3 *
Near Untrained (max = 36)	32	36	+4
Far Untrained (max = 64)	63	63	0

## Data Availability

Data can be shared upon reasonable request to the Corresponding Author and in accordance with Italian regulations for the privacy of biomedical data.

## Supplementary Materials

The following supporting information can be downloaded at: https://www.mdpi.com/article/10.3390/biomedicines13071666/s1, Table S1: Exercises used in the traditional training.

## Abbreviations

The following abbreviations are used in this manuscript:

PVDD	Protocollo di Valutazione Disartria e Disfonia
SL	Speech–Language
AOT	Action Observation Therapy
ACE-R	Addenbrooke Cognitive Examination-Revised
ES	Equivalent Score
GHS	Gordon Holmes Syndrome
SCA	Spinocerebellar Ataxia
CS	Cerebellar Syndrome
FRDA	Friedreich Ataxia
RH	Right
LH	Left
st-Vs	Supratentorial Ventricles
Fg	Focal Gliosis
Mb	Midbrain
P	Pons
Mo	Medulla Oblongata
bl-FP	Bilateral Frontoparietal
VH	Vermal Hypothropy
SS	Supra-Sylvian
FWM	Frontal White Matter
