# Supplementary material for: An Imitation-Based Treatment for Ataxic Dysarthria: A Retrospective Multiple Single-Case Study
Source: Biomedicines. 2025 Jul 8;13(7):1666. doi: 10.3390/biomedicines13071666 (PMC12292180; doi:10.3390/biomedicines13071666)
Supplement: Supplementary file 1 [file biomedicines-13-01666-s001.zip › biomedicines-3444712-supplementary.pdf]

Table S1. Exercises used in the traditional training (Nordio et al., 2018; page 28).

Respiratory and phonatory exercises	Orofacial muscles exercises	Articulation and prosody exercises
Exercises to increase diaphragmatic and respiratory functions.	LIPS: smile alternating lip protrusion(kiss); pronunciation of explosive sounds such as /pa/; opposition of lower lip on the upper lip	Repeat or read lists of possibly long and complex words that start with or contain consonants groups
Exercises to increase expiration time using natural expiration or sibilant, with or without nasalization	TONGUE: extrusion and intrusion; lateralization on right and left; up and down movement; rotation movement	Read aloud texts, sentences, or words, making sure to articulate and pronounce the words well without diminishing or increasing the rhythm of speech, with the correct punctuation and intonation
Exercise to increase phonation time by pronouncing the vocal /a/	VELAR MUSCLE: pronunciation of occlusive consonants such as /g/ or /k/	If necessary, ask patients to hyper articulate, accelerate or slow down, or exaggerate intonation
Exercise to reduce oral stiffness, by pronouncing vocal such as /e/ preceded by soft air expiration or /m/	JAW: open and close the mouth; jaw lateralization in horizontal and sagittal plan	
